# SVR achievement in triple therapy treated hepatitis C induced cirrhosis: A dual center retrospective cohort study

**DOI:** 10.1016/j.amsu.2022.104193

**Published:** 2022-07-20

**Authors:** Naukhaiz Taqi Sheikh, Muhammad Taha Shaukat, Azhar Hussain, Ahmed Ayyan, Abdullah Iqbal, Shakiba Karim, Hasan Ilyas, Kaleem Ullah, Muhammad Junaid Tahir, Muhammad Sohaib Asghar

**Affiliations:** a Lahore General Hospital, Lahore, Pakistan; b Liver Transplant and Hepatobiliary Unit, Pir Abdul Qadir Shah Jelani Institute of Medical Sciences, Gambat, Sindh, Pakistan; c King Edward Medical University, Lahore, Pakistan; d Dow University of Health Sciences–Ojha Campus, Karachi, Pakistan

**Keywords:** Hepatitis C, Hepatocellular carcinoma, Sustained viral response (SVR), Antivirals, Treatment

## Abstract

**Background and objective:**

Multiple prospective and retrospective cohort studies from the West have demonstrated the conflicting results about the efficacy of direct-acting antivirals (DAAs) by sustained virologic response (SVR) achievement rate in hepatitis C virus (HCV) infected patients. But there is limited to no data about the effectiveness of triple therapy ribavirin-sofosbuvir-daclatasvir in cirrhotic hepatitis C infected patients from Pakistan.

**Methods:**

We conducted a retrospective cohort study by retrieving records of 359 hepatitis C infected patients treated with triple therapy from two tertiary care hospitals in Pakistan [Cirrhotic = 187 (53%); non-cirrhotic = 172 (47%)] from February 18, 2018, to June 29, 2019. We lost the follow-up of 158 (44.1%) patients due to death (n = 24, 6.68%), non-responding/wrong contact number (n = 43, 9.63%), and whom consented to study but didn't complete follow-up/refused to participate in the study (n = 91, 25.34%). Only 201 (45.9%) completed follow up, and of these 87 (43.2%) were cirrhotic patients based on Liver Stiffness Index. Analysis was run by dividing groups into subgroups; who achieved SVR/who didn't achieve SVR.

**Results:**

We analyzed the data of 201 (45.9%) who completed follow-up including cirrhotic patients (n = 87, 43.2%). Mean age was 50.6 + 10.65 years. 81 (94.18%) did achieve SVR while 5 (5.81%) did not achieve SVR. Achievement of SVR was statistically associated with low platelet count, higher total bilirubin, and lower albumin (p < 0.05) while other demographic and disease-related characteristics of patients were not statistically significant (p > 0.05).

**Conclusion:**

Triple therapy (ribavirin-sofosbuvir-daclatasvir) achieves over 94% SVR in the Pakistani population, so proved to be highly effective against hepatitis C infection.

## Highlights

• Triple therapy (ribavirin-sofosbuvir-daclatasvir) achieves over 94% SVR in the Pakistani population.
• Hence, proven to be highly effective against hepatitis C infection.
• Achievement of SVR was statistically associated with low platelet count, higher total bilirubin, and lower albumin.

## 1 Introduction

Roughly 170 million people worldwide are suffered from the hepatitis C virus (HCV). According to World Health Organization (WHO), about 3% of the world's population is affected by hepatocellular carcinoma (HCC). Hepatitis C used to be treated with peg–IFN–2a, an immunomodulatory agent, before introducing direct-acting antiviral agents (DAAs). Ribavirin, an oral antiviral nucleoside analog, was added to the regimen later. DAAs target the life cycle of HCV at different stages [1]. According to a WHO source, an estimated 10 million people in Pakistan are affected by hepatitis C [2].

Two new drug regimens were approved in 2013. The first regimen contained sofosbuvir in combination with ribavirin with or without peg-IFN. The second one included simeprevir, ribavirin, and peg-IFN. These regimens are simple to use, have better tolerability and improve patient-reported outcomes (PROs) [3]. Treatment regimens containing interferon with DAAs with or without ribavirin have been used in the start. Later, interferon-free regimens were produced combining different DAAs. DAA-based regimens have well tolerability and minimal side effects [4]. Daclatasvir is an NS5A inhibitor with pan-genotypic activity, it is effective against six significant genotypes of hepatitis C. Sofosbuvir is another pan-genotypic oral NS5B inhibitor that is also effective [5]. Sofosbuvir/daclatasvir combination has a high sustained virologic response (SVR) rate in patients with genotype 1 or 4. With the addition of ribavirin, there is more increase in the SVR rate. Peg-IFN with ribavirin was the pillar of treatment before the introduction of DAAs. This regimen has many side effects and a high pill burden. Interferon free direct-acting antiviral combination regimens are the current standards of care. Patients with hepatitis C genotype 1 or 4treated with ≥2 DAAs have high rates of SVR. Treatment should be based on an interferon-free combination, including sofosbuvir along with 1–3 other DAAs plus ribavirin as advised by European Association for Study of Liver (EASL) recommendations in 2016 [5].

Behavioral and psychological barriers impede many patients with hepatitis C from initiating HCV treatment. Also, throughout HCV treatment, behavioral and psychological transformations occur as well. These include a decrease in internalized stigma related to HCV, an increase in HCV disclosure and self-care, and a new aspiration to help others affected with HCV. All these factors affect the achievement of SVR in HCV patients [6].

In this study, we tried to evaluate the effectiveness of DAAs with ribavirin triple therapy for the treatment of HCV.

## 2 Materials and methods

**Fig. 1:**
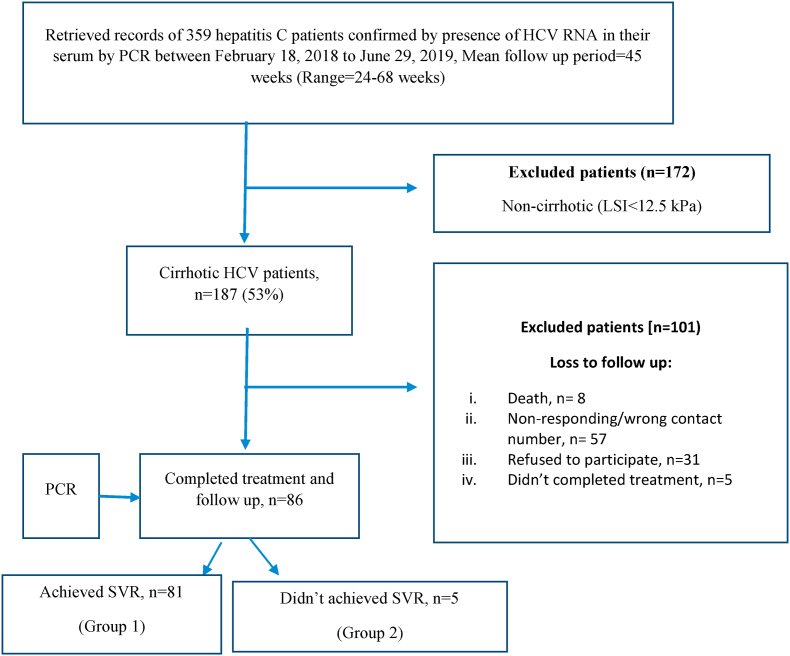
Study flow diagram.

In this retrospective cohort study, conducted in the Hepatitis Clinic of Lahore General Hospital, Lahore, and Gambat Liver Transplant Center, Sindh, Pakistan, we retrieved the records of 359 hepatitis C patients confirmed by the presence of HCV RNA in their serum by PCR. The data was collected from February 18, 2018 to June 29, 2019. The mean follow-up period for our cohort was 45 weeks after 12 (SVR_12_) weeks of completion of treatment with a minimum of 24 weeks or 6 months to counter the doubling time of HCC (Fig. 1). The absolute goal of chronic hepatitis treatment is to attain long-term viral clearance that is predicted by bringing about a sustained virologic response (SVR or SVR_12_) defined as undetectable HCV RNA in blood 12 weeks after treatment withdrawal.

Then, we excluded 172 non-cirrhotic patients which were having Liver Stiffness Index <12.5 kPa leaving behind 187 cirrhotic patients. We lost the follow-up of 101 patients; among them, 8 died during this period. 57 subjects did not provide appropriate contact numbers to be called for follow-up and 31 subjects refused to come for follow-up. While 5 patients did not complete therapy.

Only 86 patients completed therapy and came for follow-up. When they came for follow-up, they were checked by consultants of our hepatitis clinic and PCR was recommended to check the status of SVR_12_. If no HCV RNA was detected in their serum, they were placed in group 1 and labeled as ‘SVR achieved’. If HCV RNA was detected in their serum, they were placed in group 2 and labeled as ‘SVR did not achieved’. This study was registered by the institutional ethical review board of PAQSJ Institute of Medical Sciences, Gambat, Pakistan (Reference UIN: PAQSJIMS/IRB/863). Consent to participate was taken in an informed written manner. The work has been reported in line with the STROCSS criteria [7].

## 3 Statistical analysis

Mean and standard deviation were calculated for quantitative variables while Chi-square/Fischer Exact test was used for qualitative variables. Univariate analysis was done through the independent student t-test. A *p*-value < 0.05 was considered statistically significant. The data were analyzed by using SPSS version 25.

## 4 Results

Only 86 patients completed therapy and came for follow-up. 49 (56.9%) were female while 37 (43.02%) were male. The mean age was 50.6 + 10.65 years. The mean FibroScan score was 24.20 + 12.45. Other descriptive statistics of the study population and labs are given in Table 1.

**Table 1:** Descriptive statistics of study population (n = 86).

	Mean	Std. Deviation
Age (years)	50.6279	10.65728
Height (cm)	156.6582	9.62753
Weight (kg)	61.2716	15.00917
FibroScan Score (kPa)	24.2035	12.45713
Hb (g/dL)	13.1347	4.12192
RBCs (*10^6^ cells/mm^3^)	4.9499	.84505
Hct (%)	39.0567	6.88922
MCV (fL)	79.6789	7.39504
MCH (pg)	27.5986	9.17513
TLC (* 10^3^ cells/mm^3^)	8.2824	3.13760
Neutrophils (%cells/mm^3^)	58.7794	13.14678
Lymphocytes (%cells/mm^3^)	31.3154	10.69520
Monocytes (%cells/mm^3^)	3.8270	2.94280
Platelets ( × 10^11^/unit)	184.3784	93.70161
PT (seconds)	16.9543	3.57564
APTT (seconds)	35.6842	8.11440
INR	1.2108	.30414
ALT (units/L)	97.2462	68.04296
AST (units/L)	87.4308	49.24840
ALP (units/L)	330.9885	166.37542
Total Bilirubin (mg/dL)	1.1508	.80161
Serum Albumin (g/dL)	3.5517	.88090

**Abbreviations:** Hb: Hemoglobin, ALT: Alanine aminotransferase, AST: Aspartate aminotransferase, INR: International normalized ratio, RBCs: Red blood cells, Hct: Hematocrit, MCV: Mean corpuscular volume, MCH: Mean corpuscular heamoglobin, TLC: Total leukocyte count, PT: Prothrombin time, APTT: Activated partial prothrombin time, ALP: Alkaline phosphatase.

81 (94.18%) did achieve SVR while 5 (5.81%) did not achieve SVR. Achievement of SVR was statistically associated with a family history of HCV-related HCC (*p* = 0.012) while other demographic characteristics patients like the gender of the patient, history of smoking, diabetes mellitus, alcohol drinking, and HBV infection were not statistically significant (p > 0.05) (Table 2).

**Table 2:** Relationship of various patient related factors and achievement of SVR (n = 86).

	SVR achieved (n = 81)	SVR not achieved (n = 5)	*p value*
Gender			
**Male (37)**	36	1	0.35
**Female (49)**	45	4	
History of smoking			
**Yes**	27	3	0.337
**No**	54	2	
Diabetes			
**Yes**	34	4	0.165
**No**	47	1	
Alcohol Drinking			
**Yes**	3	0	0.231
**No**	78	5	
Hepatitis B Infection			
**Yes**	16	0	0.578
**No**	63	5	
Family History of HCV Related Hepatocellular carcinoma			
**Yes**	2	2	**0.012**
**No**	84	3	

Achievement of SVR was statistically associated with platelet count, total bilirubin, and albumin (p < 0.05) while other disease-related characteristics of patients like red blood cells (RBCs) count, prothrombin time, activated partial prothrombin time, International Normalized Ratio (INR), Alanine Aminotransferase (ALT), Aspartate Aminotransferase (AST), stage of fibrosis (early fibrosis versus significant fibrosis/cirrhosis), and Alkaline Phosphatase (ALP) were not statistically significant (p > 0.05) (Table 3).

**Table 3:** Relationship of various disease related factors and achievement of SVR (n = 86).

	SVR	Mean	Std. Deviation	p value
**RBCs** (*10^6^ cells/mm^3^)	Yes	4.9598	.85349	0.648
	No	4.7300	.72746	
**Platelets** ( × 10^11^/unit)	Yes	190.0000	94.55499	0.0001
	No	106.8000	13.25519	
**PT** (seconds)	Yes	17.0452	3.70676	0.582
	No	16.0000	1.63299	
**APTT** (seconds)	Yes	36.2353	8.22066	0.227
	No	31.0000	6.00000	
**INR**	Yes	1.2143	.31047	0.776
	No	1.1500	.21213	
**ALT** (units/L)	Yes	95.1667	67.98658	0.398
	No	122.2000	71.11399	
**AST** (units/L)	Yes	84.5833	48.75546	0.107
	No	121.6000	46.51129	
**ALP** (units/L)	Yes	325.9161	146.71304	0.706
	No	387.8000	338.89851	
**Total Bilirubin** (mg/dL)	Yes	1.0183	.66501	0.0001
	No	2.7400	.58138	
**Albumin** (g/dL)	Yes	3.7260	.84056	0.013
	No	2.6800	.49699	

**Abbreviations:** ALT: Alanine aminotransferase, AST: Aspartate aminotransferase, INR: International normalized ratio, RBCs: Red blood cells, ALP: Alkaline phosphatase.

## 5 Discussion

According to WHO, 3% of the world's population is affected by HCV. HCV used to be treated with peg–IFN–2a, an immunomodulatory agent, before introducing direct-acting antiviral agents (DAAs). Ribavirin, an oral antiviral nucleoside analog, was added to the regimen later. However, DAAs proved to be a revolution in the treatment and eradication of Hepatitis around and they target the life cycle of HCV at different stages [8]. According to WHO, an estimated 21 million people in Pakistan are affected by hepatitis C. Although, primary exposure to symptomatic disease development, there is a considerably large incubation period which can be targeted to eradicate the virus before it takes symptomatic form. DAAs with or without Ribavirin have been effective in achieving sustained viral response at different levels among different hepatitis types and genotypes [9].

Despite being prevailed by an uncooperative Pakistani population, we were able to complete a follow-up of 86 patients. 81 (94.18%) achieved SVR while 5 (5.81%) did not achieve SVR which indicates excellent efficacy of the triple therapy regime in the Pakistani population. It also indicated very good compliance and adherence to treatment of DAAs in the form of triple therapy. Achievement of SVR was statistically associated with a family history of HCV-related HCC development of HCC in their family members which was quite obvious that there were certain socioeconomic factors and family-run attitudes (lack of health literacy, lack of vaccination and/or screening for Hepatitis A and C and treatment non-compliance) that are causing the development of HCC more frequently in those who did not achieve SVR than those who achieved SVR, as reported in already published literature [[10], [11], [12], [13], [14]]. While other demographic characteristics of patients like the gender of the patient, history of smoking, diabetes mellitus, alcohol drinking and, HBV infection were not statistically significant as indicated by other studies [[15], [16], [17]].

Achievement of SVR was statistically associated with platelet count, total bilirubin, and albumin which again made perfect sense that there was more damage to liver caused by HCV virus and this extravagant hepatocyte insult leads to more decline in synthetic and physiologic functioning of the liver. Similarly, rate SVR achievement was not associated with the stage of fibrosis and DAAs have been highly effective irrespective of the degree of fibrosis in Hepatitis C, and similar association was observed in other studies [[18], [19], [20]]. While other disease related characteristics of patients like RBCs count, prothrombin time, activated partial prothrombin time, INR, ALT, AST, and ALP were not statistically significant (p > 0.05).

In conclusion, triple therapy achieves over 94% SVR in Pakistani population demonstrating a great immunogenic response induced by the three-drug treatment regimen with ribavirin-sofosbuvir-daclatasvir, so it is proved to be highly effective against Hepatitis C infection eradication. There are a few limitations of our study including that results may be confounded by a smaller sample size due to a high loss to follow-up rate. Similarly, retrospective study design also hampers entailing the ideal real-world scenario of SVR achievement rate with three-drug treatment regimen with ribavirin-sofosbuvir-daclatasvir. So, the studies with a much larger sample size and prospective study design are recommended to validate our results.

## 6 Conclusion

Triple therapy (ribavirin-sofosbuvir-daclatasvir) achieves over 94% SVR in the Pakistani population, so proved to be highly effective against hepatitis C infection.

## Ethical approval

Ethical approval was taken in this study from institutional ethical review board of PAQSJ Institute of Medical Sciences, Gambat, Pakistan (Reference number: PAQSJIMS/IRB/863).

## Sources of funding

None.

## Author contribution

NTS, AH and MTS conceived the idea; AA, AI and SK collected the data; AH and KU analyzed and interpreted the data; AH, KU, HS, MJT, and MSA did write up of the manuscript; and finally, MSA and MJT reviewed and revised the manuscript for intellectual content critically. All authors approved the final version of the manuscript.

## Trail registry number

1. Name of the registry: PAQSJ Institute of Medical Sciences, Gambat.

2. Unique Identifying number or registration ID: PAQSJIMS/IRB/863.

3. Hyperlink to your specific registration (must be publicly accessible and will be checked):

## Guarantor

Muhammad Sohaib Asghar.

## Provenance and peer review

Externally peer reviewed not commissioned.

## Annals of medicine and surgery

The following information is required for submission. Please note that failure to respond to these questions/statements will mean your submission will be returned. If you have nothing to declare in any of these categories then this should be stated.

## Consent

Studies on patients or volunteers require ethics committee approval and fully informed written consent which should be documented in the paper.

Authors must obtain written and signed consent to publish a case report from the patient (or, where applicable, the patient's guardian or next of kin) prior to submission. We ask Authors to confirm as part of the submission process that such consent has been obtained, and the manuscript must include a statement to this effect in a consent section at the end of the manuscript, as follows: “Written informed consent was obtained from the patient for publication of this case report and accompanying images. A copy of the written consent is available for review by the Editor-in-Chief of this journal on request”.

Patients have a right to privacy. Patients’ and volunteers' names, initials, or hospital numbers should not be used. Images of patients or volunteers should not be used unless the information is essential for scientific purposes and explicit permission has been given as part of the consent. If such consent is made subject to any conditions, the Editor in Chief must be made aware of all such conditions.

Even where consent has been given, identifying details should be omitted if they are not essential. If identifying characteristics are altered to protect anonymity, such as in genetic pedigrees, authors should provide assurance that alterations do not distort scientific meaning and editors should so note.

Consent to participate was taken in an informed written manner.

## Declaration of competing interest

None.
